# Triglyceride and non-HDL-C are better predictors of cardiovascular disease risk factors in Chinese Han children and adolescents than LDL-C

**DOI:** 10.1186/1687-9856-2013-S1-O39

**Published:** 2013-10-03

**Authors:** Wei-fen Zhu, Li Liang, Chun-lin Wang

**Affiliations:** 1 Department of Endocrinology, The Children’s Hospital of Zhejiang University School of Medicine, Hangzhou, China

## Objective

To validate serum cholesterol and triglycerides (TG) as predictors for the presence of cardiovascular disease risk factors in Chinese Han children and adolescents.

## Subject

A total of 203 simple obesity group included 153 boys and 50 girls with mean age 10.15±1.95 y and 432 obesity with metabolic abnormalities group (hypertension, elevated fasting blood glucose or dyslipidemia, diagnosis was achieved if any of them was reached) included 314 boys and 118 girls with mean age 10.75±2.20 y were studied. The controls consisted of 315 age and gender-matched healthy children including 214 boys and 101 girls with mean age 10.38 ± 2.84 y.

## Methods

Anthropometric indices were measured. Blood pressure was evaluated and hypertension was defined based on IDF 2007 definition, which systolic blood pressure(SBP) ≥130mmHg or diastolic blood pressure(DBP) ≥85mmHg. Biochemical measurements were done. Elevated fasting blood glucose(FBG) was considered as FBG ≥126 mg/dl (≥5.6 mmol/l) and dyslipidemia was defined according to Expert Panel on Integrated Guidelines for Cardiovascular Health and Risk Reduction in Children and Adolescents. Receiver operating characteristic (ROC) curves were used to analysis the detection of cardiovascular disease risk factors by serum cholesterol and TG in Chinese Han children and adolescents.

## Results

Values for SBP, DBP, FBG, low density lipoprotein cholesterol(LDL-C), non-high density lipoprotein cholesterol(non-HDL-C) and TG increased significantly with increasing obesity, whereas high density lipoprotein cholesterol(HDL-C) decreased with increasing obesity. Range of areas under ROC curves for TG and non-HDL-C was 0.80-0.86 and 0.67-0.75 to detect cardiovascular disease risk factors respectively, while the range of areas for LDL-C, TC and HDL-C was between 0.64-0.73, 0.60-0.69 and 0.30-0.38 respectively.

## Conclusions

Chinese Han children and adolescents with obesity are naturally at increased risk for hypertension, impaired glucose metabolism or dyslipidemia. Triglyceride and non-HDL-C are better predictors of cardiovascular disease risk factors in Chinese Han children and adolescents than LDL-C.

